# Supplementary material for: Non-invasive biomechanical characterization of embryos using microfluidic cantilevers
Source: Eur Biophys J. 2026 Jan 20;55(1):111–8. doi: 10.1007/s00249-026-01814-x (PMC12929246; doi:10.1007/s00249-026-01814-x)
Supplement: Supplementary file 2 — Supplementary Material 2 [file 249_2026_1814_MOESM2_ESM.pdf]

Supplementary Information

Supplementary File S1. Fitting procedure

Contact points were identified automatically in AtomicJ using the model-based estimator. We imported the data files from the Nanosurf AFM into AtomicJ for analysis. We analyzed the approach of each curve with the Paraboloid (Hertz) model (non-adhesive, linear elastic) and Poisson's ratio $\nu = 0.5$. For contact detection we used the Robust focused-grid search "Based on contact model," and fits were performed with Robust (HLTS) regression (coverage ~85–90%). This procedure determines the contact point as the position that yields the best agreement with the Hertz model while keeping the pre-contact baseline flat; the resulting contact is then used to compute indentation and fit the modulus. Because the embryo is compressed between two rigid surfaces, the total post-contact approach Δd is shared by the two contacts. Our derived dual-contact relation is (equation S1)

$$F = \frac{4}{3} E^{*} \sqrt{R} \left(\frac{\Delta d}{2}\right)^{3/2}, \qquad E^{*} = \frac{E}{1-\nu^{2}}$$

AtomicJ's Paraboloid (Hertz) form uses the standard single-contact expression (equation S2):

$$F = \frac{4}{3} E^{*} \sqrt{R_{\text{eff}}}\, \delta^{3/2}$$

where δ is the indentation variable supplied to the model.

To use full post-contact displacement as AtomicJ's indentation $\delta = \Delta d$ and still match our dual-contact physics, we set an effective radius (equation S3):

$$\left(\frac{\Delta d}{2}\right)^{3/2} = \frac{\Delta d^{3/2}}{2^{3/2}} \Rightarrow \sqrt{R_{\text{eff}}}\, \Delta d^{3/2} \equiv \sqrt{R}\, \frac{\Delta d^{3/2}}{2^{3/2}} \Rightarrow R_{\text{eff}} = \frac{R}{8}.$$

Accordingly, in AtomicJ we implemented the dual-contact geometry by specifying an effective spherical radius $R_{eff}=R/8$ while fitting the full post-contact displacement. The embryo radii were: Embryo 1: R=54.5 µm, Embryo 2: R=49.78 µm, Embryo 3: R=50.5 µm, yielding R_{eff} values of 6.81 µm, 6.22 µm, and 6.31 µm, respectively. Representative fits for each measurement are shown above.

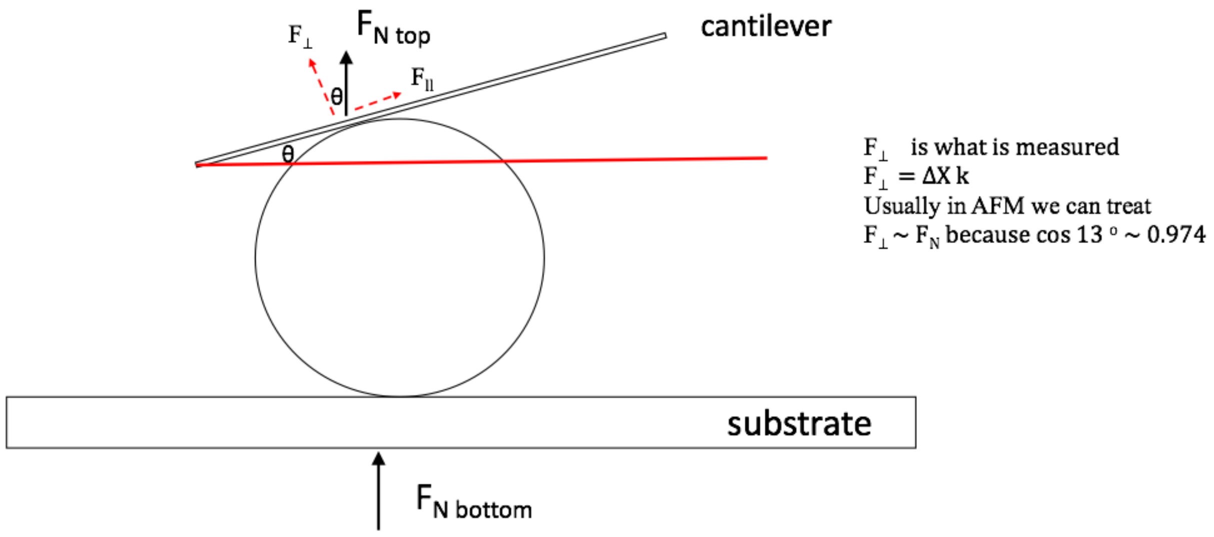

Figure S1. Schematic of the derivation. In our analysis, the measured cantilever load can be decomposed into components normal and parallel to the embryo surface. The normal component is the one that enters the Hertz model. $F\perp = F_{top} \cos \theta$ (equation S4). For geometry $\theta \approx 13°$, hence Cos $\theta \approx 0.974$. The resulting correction is therefore 2.6%. Because this is small relative to other experimental uncertainties (e.g., baseline drift, CP placement), our primary analysis treats $F\perp \approx F_{top}$. While planar probes can eliminate tilt by construction (as in L. Andolfi, S.L.M. Greco, D. Tierno, R. Chignola, M. Martinelli, E. Giolo, S. Luppi, et al., Acta Biomater. 94, 505-513), in our configuration the tilt contributes at most a minor, well-bounded systematic error and does not materially affect the fitted Young's modulus.

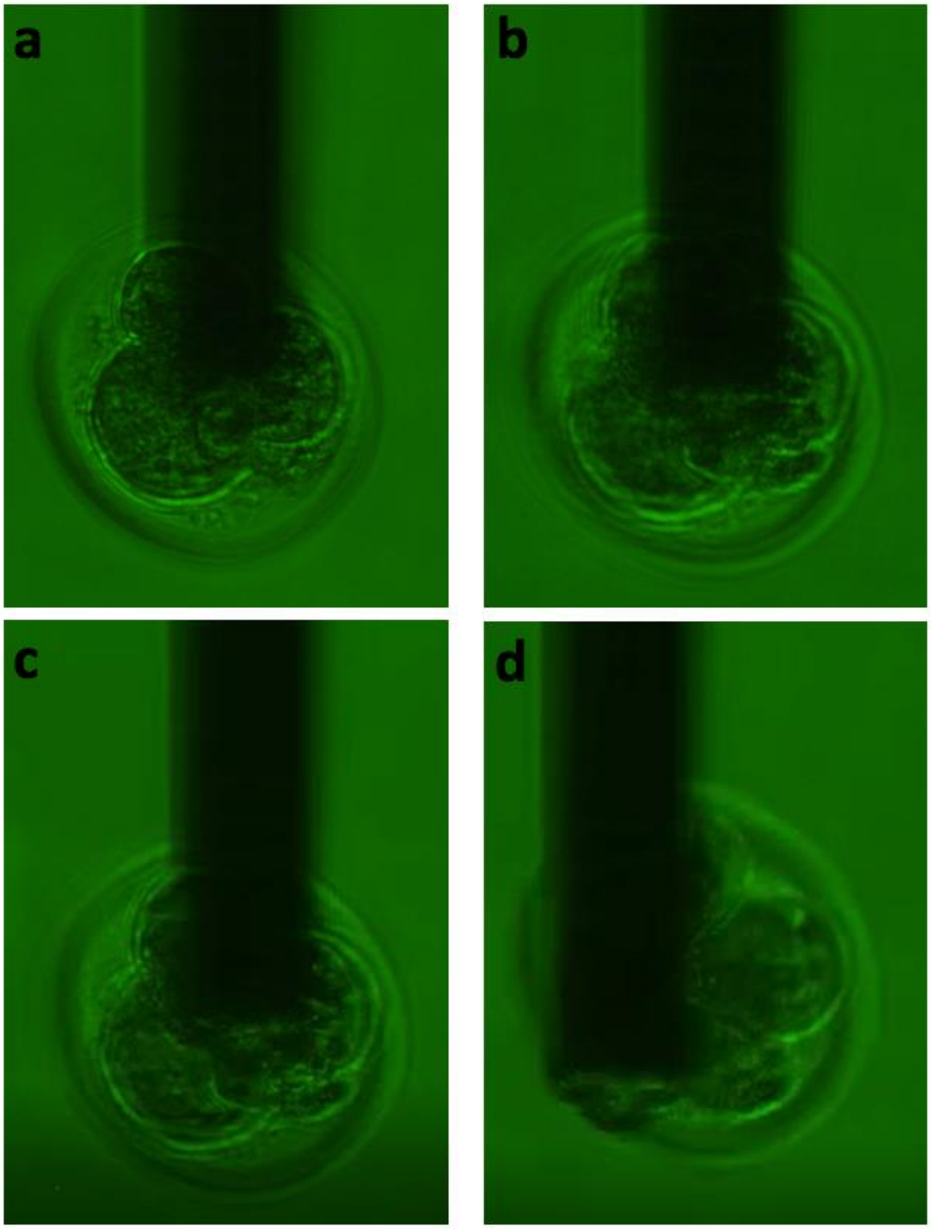

Figure S2. Time course during mechanical testing. a) At capture. **b)** After ~5 minutes of measurements. **c)** ~8 minutes after capture, a few seconds before release. **D)** Immediately after release. Bright-field images; same field of view.

Fig. S3.1 Embryo 1a

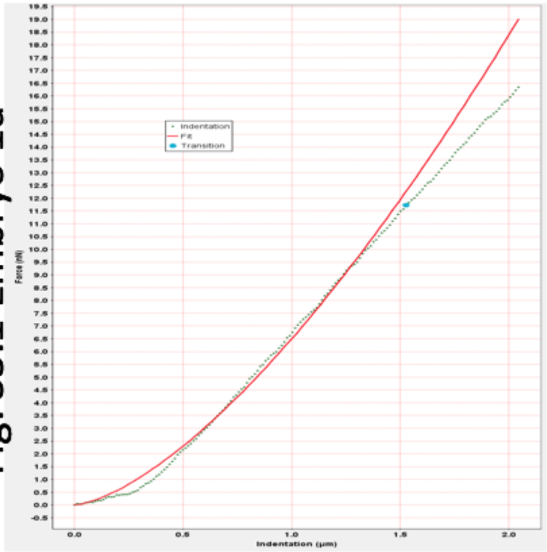

Fig. S3.2 Embryo 1b

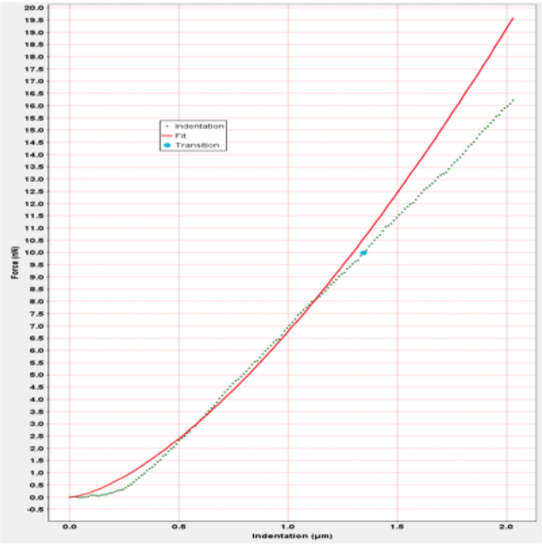

Fig. S3.3 Embryo 1c

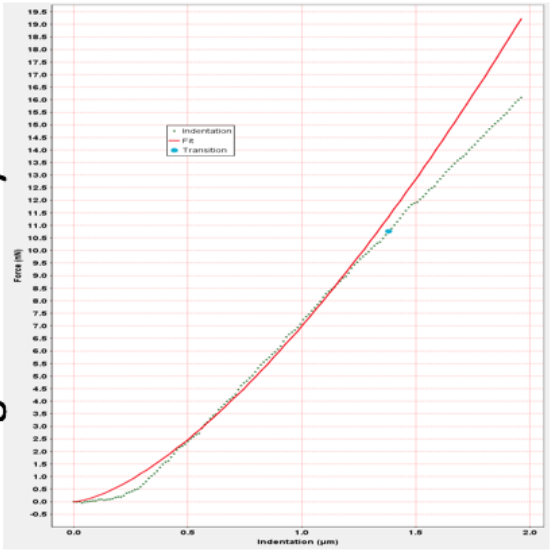

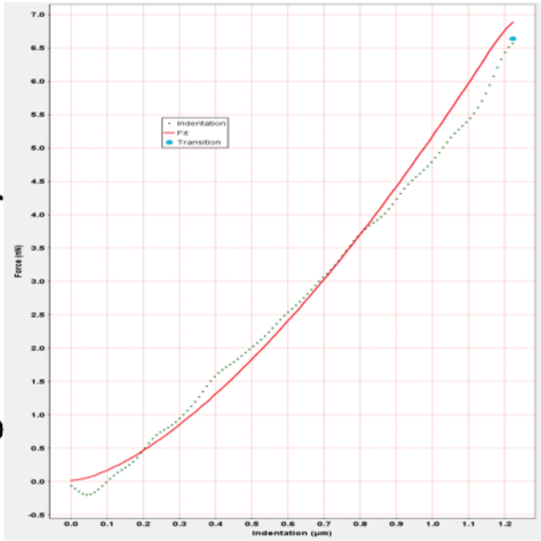

Fig. S3.4 Embryo 2a

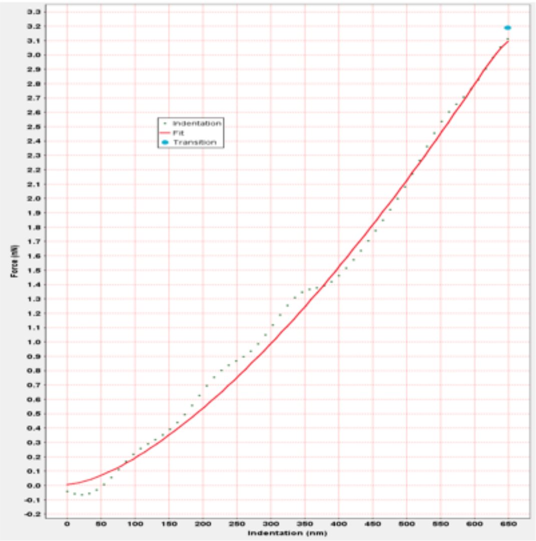

Fig. S3.5 Embryo 2b

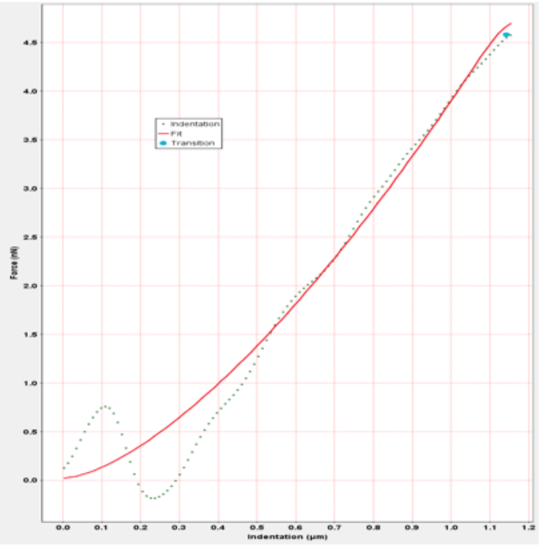

Fig. S3.6 Embryo 2c

Fig. S3.7 Embryo 3a

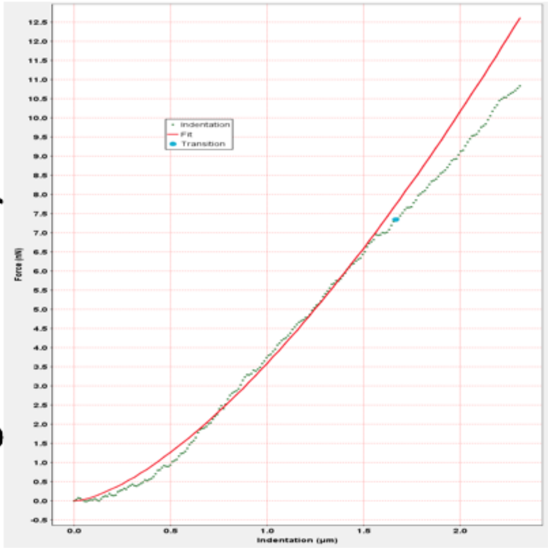

Fig. S3.8 Embryo 3b

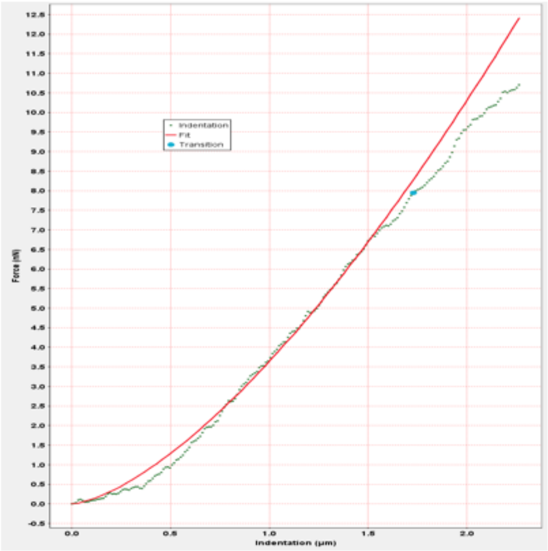

Fig. S3.9 Embryo 3c

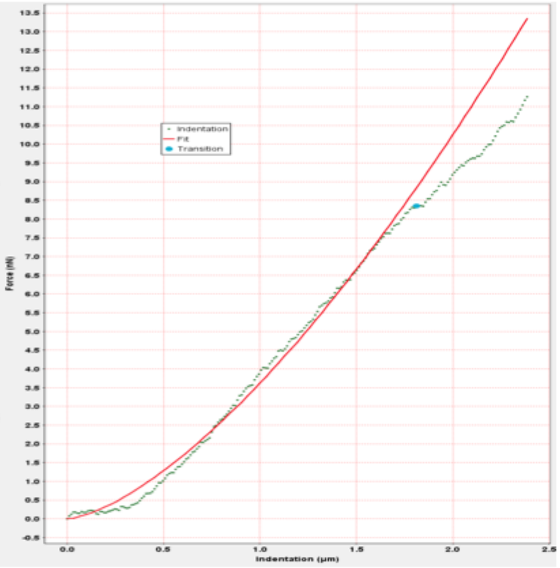

Figure S3. Indentation curves and Hertz fits. The dotted curve shows the raw indentation data; the red curve is the Hertz fit computed in AtomicJ (ν=0.5). The contact point (labeled on each panel) is identified automatically by AtomicJ's model-based estimator. Plots were exported directly from AtomicJ.

Measurement	Young’s modulus (kPa)	R^2
Embryo 1a	1.3661	0.9579
Embryo 1b	1.2696	0.9729
Embryo 1c	1.3226	0.9463
Embryo 2a	1.1718	0.9834
Embryo 2b	1.3614	0.9897
Embryo 2c	0.8823	0.9509
Embryo 3a	0.7752	0.9708
Embryo 3b	0.7863	0.98
Embryo 3c	0.782	0.9563

Table S1. Young’s modulus and R^2 values for each of the measurements. All analyses were performed in AtomicJ using the Paraboloid (Hertz) model (non-adhesive) with $\nu = 0.5$. Across all nine curves the R^2 values are uniformly high **(**range 0.946–0.990**,** mean ≈ 0.968**),** indicating excellent agreement with the Hertz model in the selected fitting window.
